# Supplementary material for: Bioengineered bacterial vesicles as biological nano-heaters for optoacoustic imaging
Source: Nat Commun. 2019 Mar 7;10:1114. doi: 10.1038/s41467-019-09034-y (PMC6405847; doi:10.1038/s41467-019-09034-y)
Supplement: Supplementary file 1 — Supplementary Information [file 41467_2019_9034_MOESM1_ESM.pdf]

Supplementary Information

Bioengineered bacterial vesicles as biological nano-heaters for optoacoustic imaging

Gujrati et al.

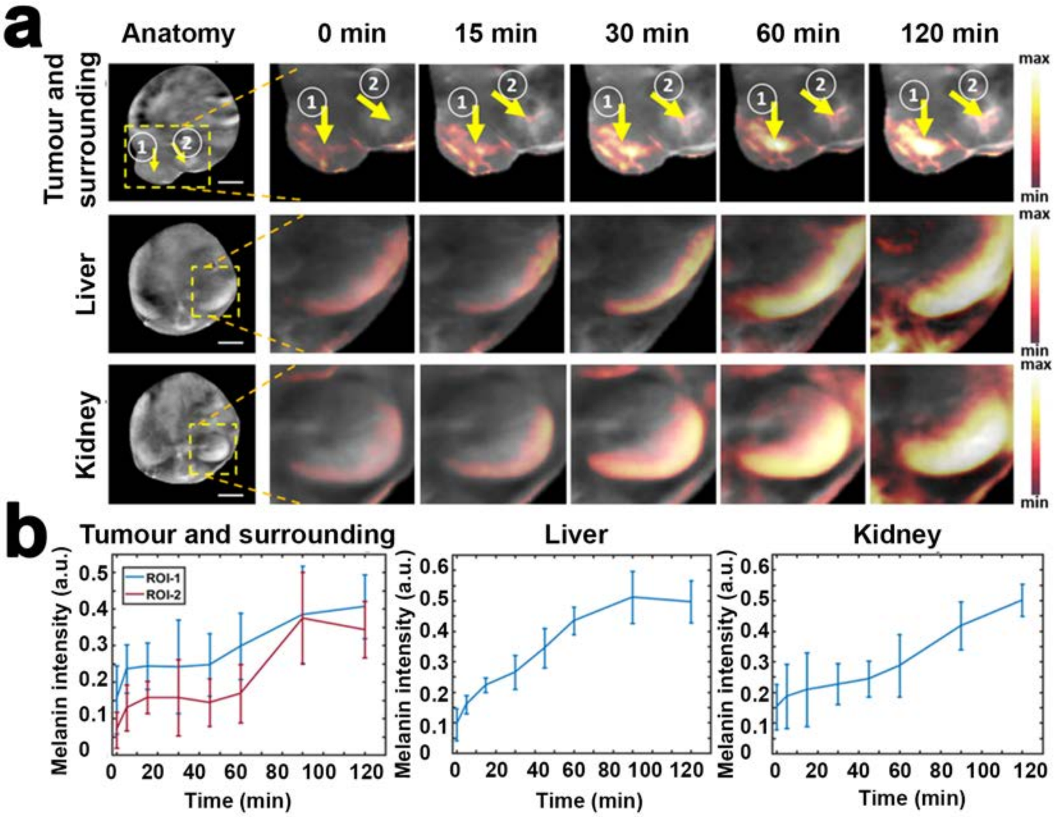

Supplementary Figure 1. In vivo MSOT imaging. (a) Melanin signal was monitored in the tumour, surround region, and in vital organs (liver and kidney) during 2 h after tail vein injection of OMV^{Mel} in a mouse. The arrow indicates an ROI in the tumor (ROI-1) and surrounding region (ROI-2). Scale bar, 5 mm. **(b)** Melanin intensity was evaluated in the tumour (ROI-1), surrounding region (ROI-2), liver and kidney, in time dependent manner (0 to 120 min). Melanin intensities are averages calculated from 6 adjacent slices from the tumour centre.

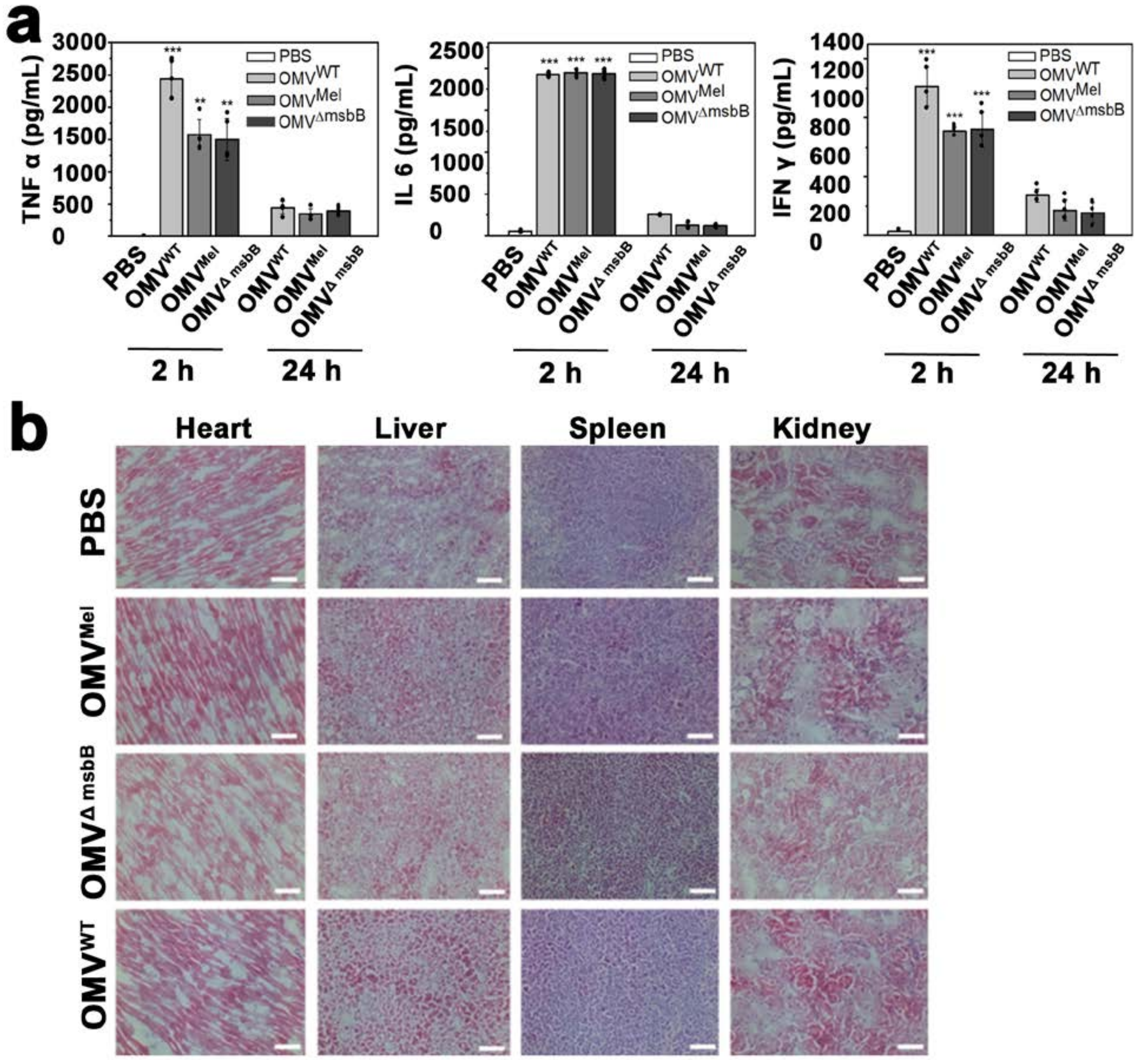

Supplementary Figure 2. In vivo immunogenicity and safety. (a) Serum levels of TNF-α, IL-6 and IFN-γ were quantified at 2 and 24 h after i.v injection of PBS, OMV^{WT}, OMV^{Mel} or $OMV^{\Delta msbB}$. Mean values and error bars are presented as mean ± SD, inter-group differences were assessed for significance using the paired t test compared to control (***$p < 0.001$ vs. PBS; n=5 mice/group). **(b)** Histology of heart, liver, spleen and kidney extracted at 24 h after injection of PBS, OMV^{WT}, $OMV^{\Delta msbB}$, or OMV^{Mel}. Scale bar, 50 μm.
